# Facile Synthesis of Polypyrrole-Functionalized CoFe_2_O_4_@SiO_2_ for Removal for Hg(II)

**DOI:** 10.3390/nano9030455

**Published:** 2019-03-19

**Authors:** Yuhao Zhao, Kai Xia, Zhenzong Zhang, Ziming Zhu, Yongfu Guo, Zan Qu

**Affiliations:** 1 School of Environmental Science and Engineering, Suzhou University of Science and Technology, Suzhou 215011, China; 1713022014@post.usts.edu.cn (Y.Z.); 1713022009@post.usts.edu.cn (K.X.); 1713022013@post.usts.edu.cn (Z.Z.); 1513022025@post.usts.edu.cn (Z.Z.); 2 Jiangsu Provincial Key Laboratory of Environmental Science and Engineering, Suzhou University of Science and Technology, Suzhou 215011, China; quzan@sjtu.edu.cn; 3 School of Environmental Science and Engineering, Shanghai Jiao Tong University, Shanghai 200240, China

**Keywords:** adsorbent, polypyrrole, heavy metal, mercury, modification

## Abstract

In order to avoid using toxic or harmful operational conditions, shorten synthesis time, enhance adsorption capacity, and reduce operational cost, a novel magnetic nano-adsorbent of CoFe_2_O_4_@SiO_2_ with core–shell structure was successfully functionalized with polypyrrole (Ppy). The physical and chemical properties of CoFe_2_O_4_@SiO_2_-Ppy are examined by various means. The as-prepared CoFe_2_O_4_@SiO_2_-Ppy nanomaterial was used to adsorb Hg^2+^ from water. During the process, some key effect factors were studied. The adsorption process of Hg^2+^ onto CoFe_2_O_4_@SiO_2_-Ppy was consistent with the pseudo-second-order kinetic and Langmuir models. The Langmuir capacity reached 680.2 mg/g, exceeding those of many adsorbents. The as-prepared material had excellent regeneration ability, dispersibility, and stability. The fitting of kinetics, isotherms, and thermodynamics indicated the removal was endothermic and spontaneous, and involved some chemical reactions. The application evaluation of electroplating wastewater also shows that CoFe_2_O_4_@SiO_2_-Ppy is an excellent adsorbent for Hg^2+^ ions from water.

## 1. Introduction

Currently, with the enlargement of industrial production, an enormous amount of wastewater is discharged into water bodies, causing serious of pollution of soil, air, water, and other issues. Especially, the pollution of heavy metals resulting from industrial wastewater is increasingly severe [1]. Among them, mercury pollution is a unique issue, due to its highly toxicity, easy migration, and bioaccumulation in human beings [2,3]. Therefore, research on mercury removal is being actively carried out all over the world. In order to remove mercury from aqueous solution, adsorption technology, as a cost-effective method, is widely researched and used [4]. Among many adsorbents, magnetic nanomaterials are a novel functional material type with unique physical and chemical properties, the most critical of which is that it can easily achieve separation from water under an external magnet [5], which can significantly reduce the operational cost.

However, magnetic nanomaterials, such as Fe_3_O_4_, CoFe_2_O_4_, and MnFe_2_O_4_, have poor adsorption and selectivity for heavy metals in water [6,7,8]. The poor adsorption capacity is either inherent or due to distinctive characteristics. In addition, easy agglomeration and the absence of surface active functional groups also limit their adsorption properties. Fortunately, the properties of magnetic nanomaterials can be generally improved after modification [9,10]. Thus, more and more magnetic nanomaterials are being modified and then employed to remove various pollutants, including heavy metal ions. CoFe_2_O_4_, a common magnetic nanomaterial, has the advantages of low toxicity and easy preparation and separation, and can be modified to not only improve its dispersibility in aqueous solution, but to also greatly enhance its stability.

A common coating modification is to coat CoFe_2_O_4_ particles with another coating layer (SiO_2_, C or organodisulfide polymer, etc.) under the outer layer of CoFe_2_O_4_ particles [8,11]. The good dispersibility and stability of CoFe_2_O_4_ particles in water can be realized by coating modification. However, high-efficiency removal for heavy metal mercury cannot be achieved by coating modification alone. Therefore, the surface chemical performance of CoFe_2_O_4_ still needs to be further decorated to enhance the mercury adsorption ability. Grafting modification is a good method to improve the surface chemical performance of CoFe_2_O_4_ nanomaterials. Common grafting groups include -NH_2_ [8,10], -SH [12,13], and others [14]. However, most of modification methods with -NH_2_ and -SH usually are either too complicated or use toxic, harmful, or hazardous acetone [15] and toluene [16] as reaction media [17,18]. In addition, some literature employed nitrogen protection or high temperature to obtain grafting groups [7,8]. The above grafting methods are beneficial to increasing the removal ability for mercury ions, but unfortunately greatly increase the disposal cost. Moreover, it is more likely to cause secondary pollution. In order to avoid using toxic, harmful, or hazardous solvents, it is necessary to seek a safer and more economical material.

Polymers have been widely employed in materials science. Polymers can form complexes with other materials. During the process of forming complexes, some special functional groups can be introduced to carriers. Among the polymers, polypyrrole (Ppy) has the benefits of easy large-scale preparation, excellent stability, and low preparation cost [19]. It has been widely applied in many fields such as energy memory, drug transport, and super capacitors, etc. Ppy polymerizes from pyrrole monomers under the action of oxidizing agents and can encapsulate many materials. The presence of amine in the polymer backbone allows Ppy to be used as a favorable modifier. Based on our previous research, the magnetic graphene oxide grafted with Ppy had very high removal capacity for mercury (II) ions. The Langmuir capacity reached 400 mg/g at pH 7 [20].

Hence, in the present research, a novel nanomaterial (CoFe_2_O_4_@SiO_2_-Ppy) with a core–shell structure was successfully synthesized through grafting with Ppy and was used to remove Hg^2+^ from water. The aim is to enhance the removal ability dispersibility, and stability of CoFe_2_O_4_ in water through optimizing its surface performance with a safe, economical, and facile synthesis method. Moreover, some key influence factors, including regeneration, were investigated. Meanwhile, the adsorption mechanism for Hg^2+^ was also investigated through a series of kinetic and equilibrium models.

## 2. Materials and Experimental Methods

### 2.1. Chemicals and Materials

Pyrrole (Py), sodium dodecyl benzene sulfonate (SDBS), cobaltous nitrate hexahydrate (CNH), iron acetylacetonate, ethylene glycol (EG), iron chloride hexahydrate (FeCl_3_·6H_2_O), sodium acetate anhydrous (CH_3_COONa), polyethylene glycol, cetyltrimethylammonium bromide (CTAB), ammonia water (NH_3_.H_2_O, 25–28 wt.%), and tetraethyl silicate (TEOs) were all obtained from Aladdin Reagent (Shanghai, China). The chemicals were all analytical grade.

### 2.2. Preparation of Materials

CoFe_2_O_4_@SiO_2_ was prepared based on our previous research report [13]. Briefly, a homogeneous solution with CNH (2.18 g), iron acetylacetonate (5.29 g), CH_3_COONa (6.51 g), polyethylene glycol (2.0 g) and EG (90 mL) was placed in an autoclave (150 mL) to undergo a hydrothermal reaction at 453 K for 14 h, and then CoFe_2_O_4_ nanoparticle was generated.

CoFe_2_O_4_ (0.30 g) was dispersed in CTAB solution (0.15 g CTAB, 150 mL pure water) with sonication for 20 min. TEOs (1.0 mL) and NH_3_·H_2_O (1.3 mL) are dropped into the above reaction system with mechanical stirring at 353 K for 3 h. Obtained materials were washed and then put into a muffle furnace and then calcined at 673 K for 4 h to obtain CoFe_2_O_4_@SiO_2_ nanoparticles.

A certain amount of CoFe_2_O_4_@SiO_2_ (0.15 g) and SDBS (0.025 g) were dissolved in 100 mL pure water with ultrasound treatment for 30 min and mechanical stirring was applied for 30 min. after that, 0.25 mL pyrrole solution was added slowly.

**Figure 1:**
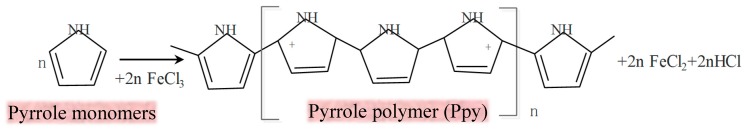
Formation scheme of Ppy with pyrrole monomers.

Subsequently, 10 mL of completely dissolved FeCl_3_·6H_2_O (3.0 g) was slowly added. The system reacted with mechanical stirring for 4 h. The Resulting product (CoFe_2_O_4_@SiO_2_-Ppy) was rinsed 3 times and then desiccated at 338 K. The formation scheme of the pyrrole polymer is shown in Figure 1.

Figure 1 shows the polymerization scheme of pyrrole monomers to produce polypyrrole polymer. It can be seen that the byproduct hydrochloric acid is produced during the progress of reaction, which increases the acidity of the reaction medium. If CoFe_2_O_4_ is directly modified with Ppy, the nature of CoFe_2_O_4_ is bound to be greatly impacted during the course of the reaction. The reason is that magnetic CoFe_2_O_4_ has a cubic spinel structure [21] and easily agglomerates and can suffer from acid corrosion. Thus, the formation of silicon shells on the surface of CoFe_2_O_4_ by hydrolysis of TEOs has a protective effect [8].

### 2.3. Sample Characterizations

The values of surface area (BET) were decided by N_2_ adsorption-desorption isotherms (Micromeritic TriStarII 3020, Norcross, GA, USA). The morphology was observed by scanning electron microscope (SEM) (FEI, Phenom, Hillsboro, OR, USA) and transmission electron microscopy (TEM) (JEM-2100F, Tokyo, Japan). X-ray Diffraction (XRD, D8 Advance, Bruker, Karlsruhe, Germany) analysis was applied to investigate the crystallization and phase. Functional groups were identified by Fourier transform infrared (FT-IR) spectrophotometry (Thermo, Nicolet-6700, Waltham, MA, USA). Magnetic strength was compared by vibrating sample magnetometry (VSM) (Quantum design, PPMS-9, San Diego, CA, USA). Elements compositions were confirmed by energy-dispersive spectrometry (EDS) and X-ray photoelectron spectroscopy (XPS) (Thermo Scientific, 250Xi, Waltham, MA, USA). The concentration of Hg^2+^ ions at any time *t* (min) was quantified using ICP-OES.

### 2.4. Batch Experiments

The solution containing a certain concentration of Hg^2+^ was prepared based on previous research report [13]. The adsorption capacities of CoFe_2_O_4_@SiO_2_-Ppy for Hg^2+^ were evaluated by initial solution pH, dosage, reaction time (*t*, min), solution temperature (*T*, K), and coexisting ions in the solution.

The effects of pH were evaluated by adding 0.1 mol/L hydrochloric acid and 0.1 mol/L sodium hydroxide solutions to adjust pH from 3 to 9. The test was performed for 8 h at 298 K by a 250 mL sealed conical flask with 100 mL Hg^2+^ solution and 5 mg adsorbent. The initial concentration (*C*_0_) of Hg^2+^ was 40 mg/L.

The effect of adsorbent dosage was investigated by adding various adsorbents of 0.03, 0.05, 0.08, 0.1, and 0.15 g/L with *C*_0_ = 40 mg/L, pH = 8 and *T* = 298 K. Contact time *t* was investigated at various time intervals of 3, 4, 5, 8, 10, 15, 30, 60, 90, 120, 240, 360, 480, 600, and 720 min with *C*_0_ = 40 mg/L, dosage of 5 mg, pH = 8 and *T* = 298 K. Isotherms were investigated at 298 K, pH = 8, and *t* = 8 h with *C*_0_ of 20, 30, 40, 50, 60, 80, and 100 mg/L.

The equilibrium capacity (*q*_e_, mg/g) was investigated according to *C*_0_, equilibrium capacity (*C*_e_, mg/g), dosage (g), and solution volume (L) [20]. The instantaneous capacity (*q*_t_, mg/g) was investigated according to *C*_0_, instantaneous concentration *C*_t_ (mg/L), dosage (g) and solution volume (L) at any time (*t*, min) [13]. The removal efficiency (*E*, %) of Hg^2+^ ions was obtained based on initial concentration *C*_0_ and equilibrium concentration *C*_e_.

## 3. Results and Discussion

### 3.1. Characterization of Materials

**Figure 2:**
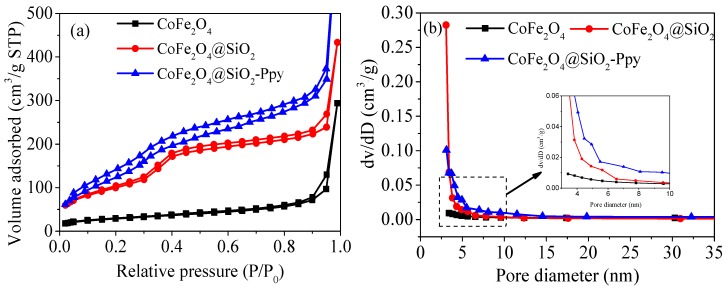
Adsorption-desorption plots (**a**); Size distribution (**b**).

Figure 2 reveals that pore diameters of CoFe_2_O_4_, CoFe_2_O_4_@SiO_2_, and CoFe_2_O_4_@SiO_2_-Ppy only changed slightly after modification with the Barrett–Joyner–Halenda method. The values of both BET and the total pore volume of as-prepared CoFe_2_O_4_@SiO_2_-Ppy significantly increased to 218.56 m^2^/g and 0.888 cm^3^/g, which are 4.5 and 2 times as large as CoFe_2_O_4_, respectively. The results not only show that the silicone shell was successfully wrapped on the outer surface of CoFe_2_O_4_, but also are conducive to enhancing the adsorption capacity of CoFe_2_O_4_@SiO_2_-Ppy.

In addition, the calcination of surfactant CTAB makes CoFe_2_O_4_@SiO_2_ exhibit a porous fluffy morphology. The calculated values of BET, total pore volume, and pore diameter data are listed in Table 1.

A noteworthy point is that the BET value of CoFe_2_O_4_@SiO_2_-Ppy is reduced compared to CoFe_2_O_4_@SiO_2_. This owes to the fact that lots of the chain-like Ppy packed on the surface of the material and sealed the porous mesh structure of the material during the continuous process of polymerization [22].

**Figure 3:**
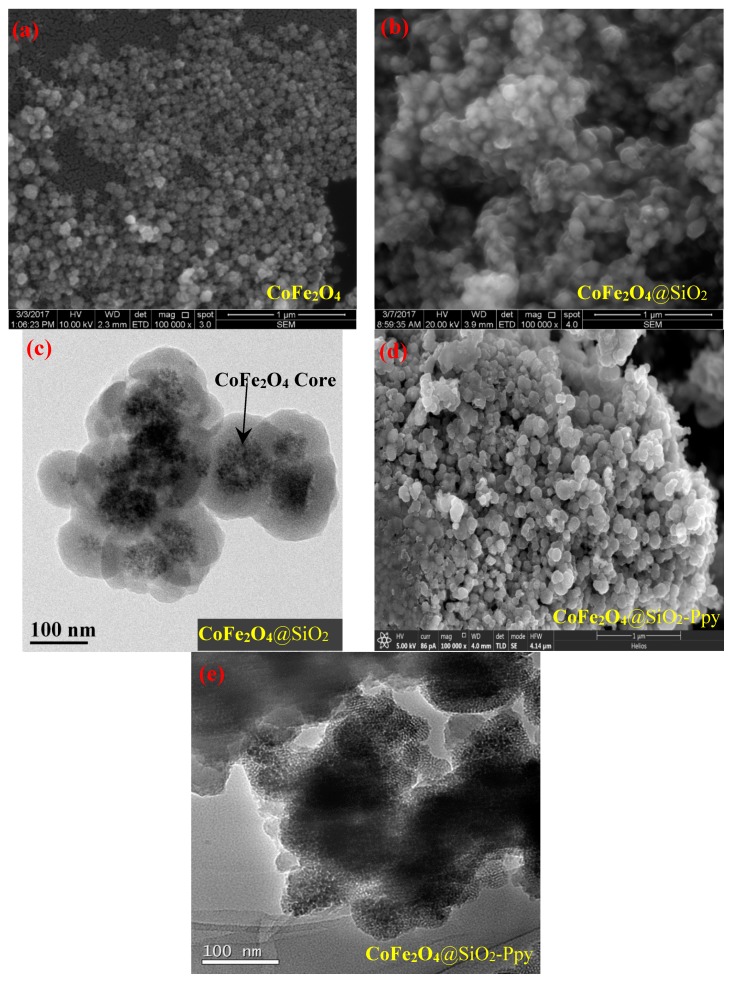
Scanning electron microscopy (SEM) of (**a**,**b**); Transmission electron microscopy (TEM) of (**c**–**e**) of the three as-prepared materials.

Figure 3 is the SEM and TEM patterens of CoFe_2_O_4_, CoFe_2_O_4_@SiO_2_, and CoFe_2_O_4_@SiO_2_-Ppy. The corresponding particle diameters are about 50–90, 70–120, and 90–140 nm, respectively. For CoFe_2_O_4_, the reason of agglomeration may be mainly due to the magnetic dipole–dipole interaction [23].

As shown in Figure 3b, the size of CoFe_2_O_4_@SiO_2_ becomes larger compared to CoFe_2_O_4_, proving that the silicon shell has successfully loaded on the surface of CoFe_2_O_4_ nanoparticles. However, most of the particles are still stuck together, resulting in a poor dispersibility. As shown in Figure 3c, the agglomeration of CoFe_2_O_4_@SiO_2_-Ppy decreases significantly, and it can be clearly observed that the material has a smooth surface and a ball-like shape. From the TEM image of CoFe_2_O_4_@SiO_2_-Ppy shown in Figure 3d, it can be judged by different electron penetration: the black core is CoFe_2_O_4_; the lighter shell is a silicon shell (SiO_2_ layer) and Ppy. The above results suggest that CoFe_2_O_4_@SiO_2_-Ppy has a core–shell structure and that CoFe_2_O_4_@SiO_2_ has been enclosed into the Ppy matrix [24].

**Figure 4:**
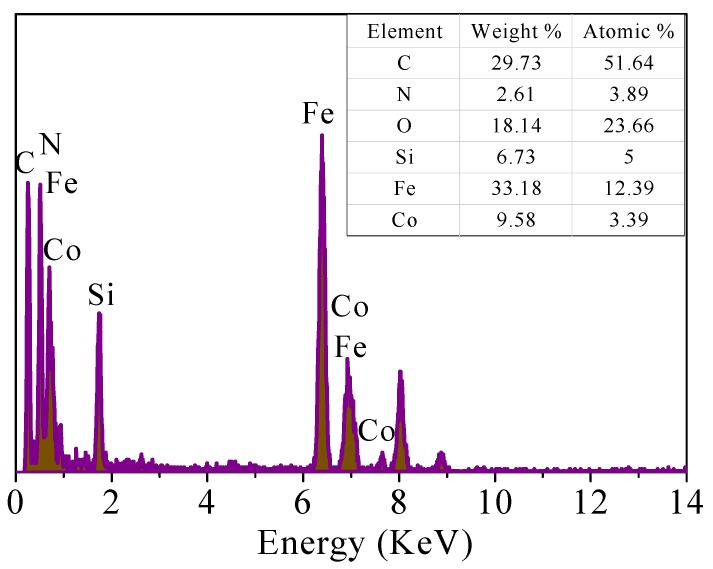
Energy-dispersive spectrometry (EDS) elemental analysis of CoFe_2_O_4_@SiO_2_-Ppy.

**Figure 5:**
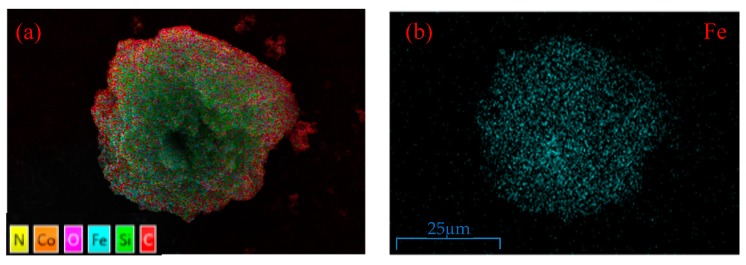

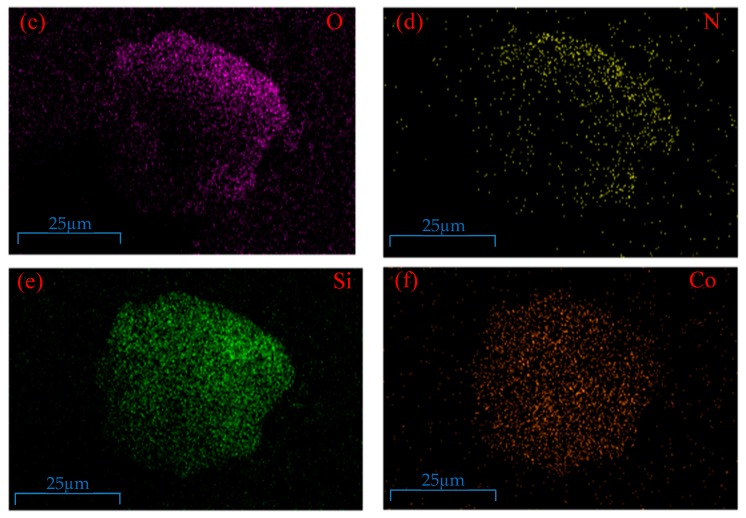
(**a**) SEM with X-ray area scanning; EDS mappings of (**b**) Fe, (**c**) O, (**d**) N, (**e**) Si, and (**f**) Co of CoFe_2_O_4_@SiO_2_-Ppy.

As shown in Figure 4, the EDS elemental analysis of CoFe_2_O_4_@SiO_2_-Ppy shows the peaks of Co, Fe, C, Si, and N and indicates the major constituents of magnetite, silice shell, and Ppy, which verifies the existence of CoFe_2_O_4_, silice shell (SiO_2_ layer), and Ppy. From Figure 5a–f, the material mainly contains Fe, N, O, Si, and Co. It shows that Ppy was successfully combined with CoFe_2_O_4_@SiO_2_ and evenly distributed on the outer surface.

**Figure 6:**
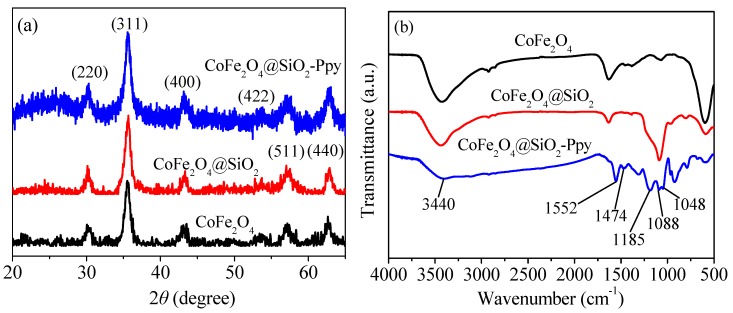
X-ray diffraction (XRD) images (**a**) and Fourier transform infrared (FT-IR) spectra (**b**).

Figure 6a shows the XRD images of as-prepared materials. For CoFe_2_O_4_, the main six peaks correspond to the (220), (311), (400), (422), (511), and (440) planes [10,13], respectively.

The characteristic peaks in Figure 6a of the three as-prepared materials were accordant with the diffraction pattern of CoFe_2_O_4_ (JCPDS No. 22-1086) [25]. The diffraction peaks of CoFe_2_O_4_@SiO_2_ covered by a silicon shell are consistent with those of CoFe_2_O_4_, and the decrease in vibration intensity may be due to the influence of the encapsulated silicon shell.

No new peaks indicate that the crystal form of the material is not be affected by the modification. In the pattern of CoFe_2_O_4_@SiO_2_-Ppy, there is a wide peak at 2*θ* of about 25°, which is the characteristic peak of Ppy [26,27], probably due to a certain level of Ppy crystallization.

According to Figure 6b, a wide peak around at 3440 cm^−1^ can be found due to tensile vibration of the surface adsorbing -OH in water [28]. The wide peak at 1088 cm^−1^ can be ascribed as Si–O–Si, indicating that there is successful attachment of a silicon shell (SiO_2_ layer) on the outer surface of CoFe_2_O_4_ [29].

A bond at 1552 cm^−1^ is the proof of the existence of Ppy, corresponding to C=C vibration [30]. Peaks at 1185, 1048, and 474 cm^−1^ are C–H stretching in plane [31], C–H bending mode vibration in plane [32,33], and the vibration of C–N in a pyrrole ring [34]. The presence of these above functional group peaks indicates that Ppy is indeed present in the CoFe_2_O_4_@SiO_2_-Ppy composite.

**Figure 7:**
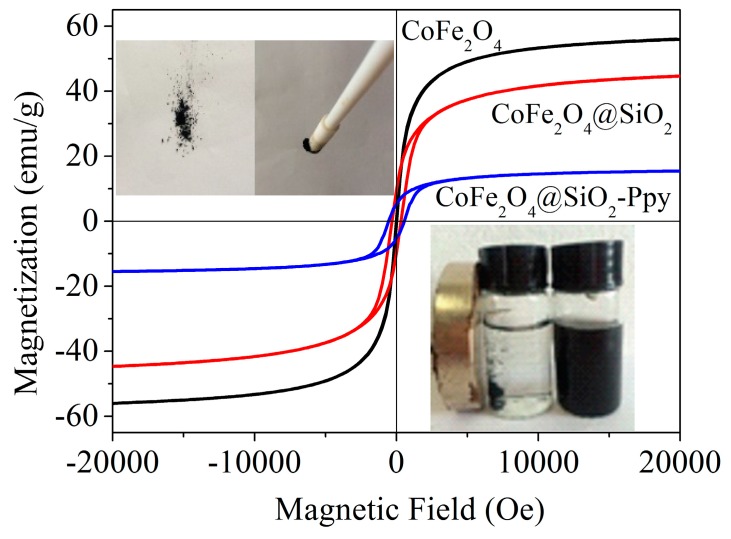
Vibrating sample magnetometry (VSM) analysis of the three as-prepared materials.

The magnetic property of each material was detected by VSM, and the corresponding analytical data was plotted in Figure 7. Based on the hysteresis loop, the saturation magnetic moments are found to be 56.03, 44.68, and 15.46 emu/g. The magnetic reduction of CoFe_2_O_4_@SiO_2_ may because of the nonmagnetic silicon shell wrapped outside the magnetic core, which also indirectly demonstrates that the silicon shell has wrapped successfully.

After Ppy loaded on the surface of CoFe_2_O_4_@SiO_2_, the magnetic value has a significant decline from 44.7 to 15.5 emu/g. The reason is probably that a small number of magnetic particles in the composite are shielded by conductive Ppy [22,35]. Although the magnetic value of CoFe_2_O_4_@SiO_2_-Ppy is weak, it can still separate quickly from the water by an outer magnetic field. The inserted picture shows the effect of magnetic separation with an outer magnet.

**Figure 8:**
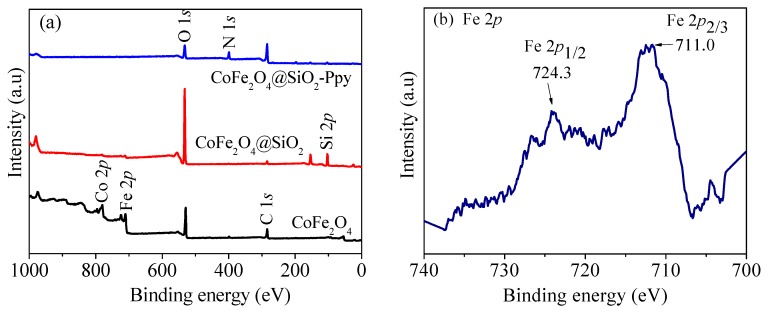

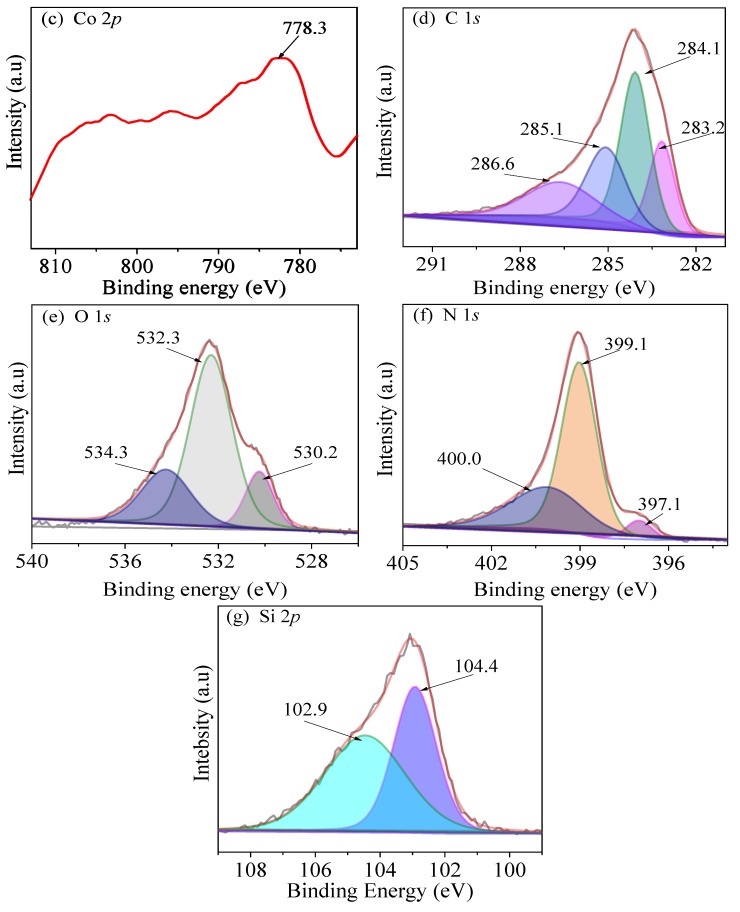
X-ray photoelectron spectroscopy (XPS) spectra of (**a**) survey scan; (**b**) Fe 2*p*, (**c**) Co 2*p*, (**d**) C 1*s*, (**e**) O 1*s*, (**f**) N 1*s*, and (**g**) Si 2*p* of CoFe_2_O_4_@SiO_2_-Ppy.

From the wide-scan XPS spectra shown in Figure 8a, it can be seen that there are six peaks at 110.1, 293.7, 405.4, 541.2, 738.2, and 813.9 eV, attributed to Si 2*p*, C 1*s*, N 1*s*, O 1*s*, Fe 2*p*, and Co 2*p*, respectively. In the pattern of CoFe_2_O_4_@SiO_2_, the peaks of Co, Fe, and C become weak and a new peak of Si 2*p* appears at 110.1 eV.

Figure 8b,c indicates successful synthesis of CoFe_2_O_4_ nanoparcicles in the as-prepared composite [13,20]. In Figure 8d, there are four C 1*s* peaks at 283.2, 284.1, 285.1, and 286.6 eV. The peaks at 284.1 and 285.1 eV are mainly attributed to *β*-carbons and *α*-carbons, respectively. The peak at 286.6 eV is assigned to C=N bonds [22,36].

The O 1*s* spectrum shown in Figure 8e has three peaks at 530.2, 532.3, and 534.3 eV. The peak at 530.2 eV is the oxygen in carbonyl group [13]. The peaks at 532.3 and 534.3 eV are related to the oxygen atoms in hydroxyl ions and water [10].

On the pattern of CoFe_2_O_4_@SiO_2_-Ppy, the N 1*s* peaks shown in Figure 8f at 397.1, 399.1, and 400.0 eV are related to NH-, -N=, and N^+^, respectively [37]. The appearance of new peaks of N 1*s* indicates the successful polymerization of pyrrole monomers. The peaks of Si 2*p* in Figure 8g are located at 102.9 and 104.4 eV, proving that a silicon shell is formed on the surface of CoFe_2_O_4_ through TEOs hydrolysis.

**Figure 9:**
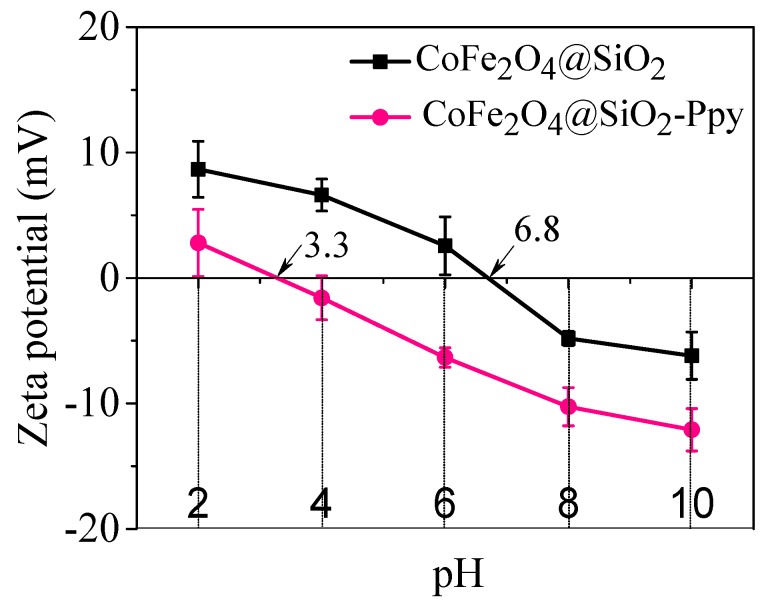
Zeta potentials of CoFe_2_O_4_@SiO_2_ and CoFe_2_O_4_@SiO_2_-Ppy.

It can be seen from the Figure 9 that the value of zero charge (pH_zc_) of CoFe_2_O_4_@SiO_2_ is 6.8. When the pH is more than 6.8, the Zeta potential of CoFe_2_O_4_@SiO_2_ is negative, indicating that SiO_2_ has been coated on the surface of CoFe_2_O_4_ [12]. However, the small Zeta potential at pH 8 indicates that the CoFe_2_O_4_@SiO_2_ solution has a poor stability.

After grafting with Ppy, the value of pH_zc_ of CoFe_2_O_4_@SiO_2_-Ppy is decreased to 3.3, which owes to the existence of -NH_2_ [38], proving a successful synthesis of CoFe_2_O_4_@SiO_2_-Ppy. In addition, the Zeta potential of CoFe_2_O_4_@SiO_2_-Ppy is −12.1 mV at pH 8, which is far lower than that of CoFe_2_O_4_@SiO_2_ of −6.2 mV. The result reveals that the solution of CoFe_2_O_4_@SiO_2_-Ppy is relatively stable, which is consistent with the data shown in Figure 3c. The results of low Zeta potential value and good stability are conducive to alleviating the agglomeration of adsorbent solution and enhancing the removal ability for positively charged Hg^2+^ ions.

### 3.2. Adsorption Performance Test

#### 3.2.1. Influence of pH

**Figure 10:**
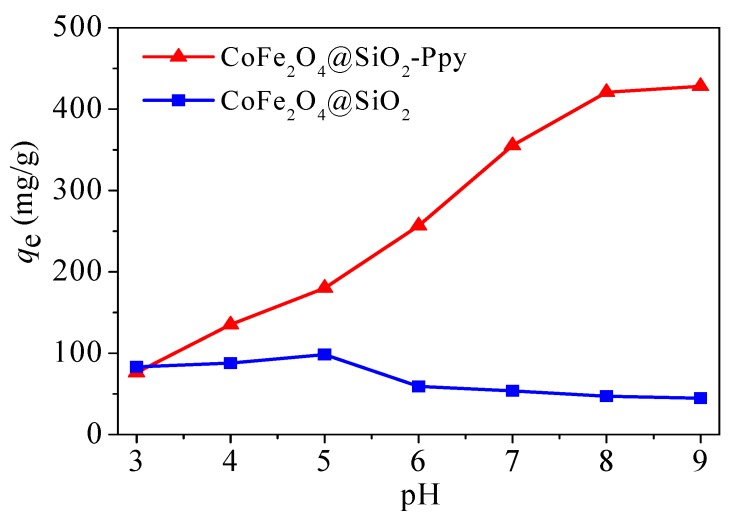
Effect of pH on removal capabilities of CoFe_2_O_4_@SiO_2_ and CoFe_2_O_4_@SiO_2_-Ppy.

It is well known that pH can affect the surface charges of adsorbents and the form of heavy metals [39]. As shown in Figure 10, CoFe_2_O_4_@SiO_2_ has low adsorption capacity for mercury ions, only 98.4 mg/g at pH = 5. Compared to CoFe_2_O_4_@SiO_2_, the adsorption capacity of CoFe_2_O_4_@SiO_2_-Ppy is greatly enhanced, and the adsorption plot has a rapid ascending tendency with the increasing pH. A basic equilibrium adsorption is reached at pH = 8 and achieves 420.8 mg/g. Therefore, pH = 8 was chosen as the reaction condition in later study.

#### 3.2.2. Influence of Dosage

**Figure 11:**
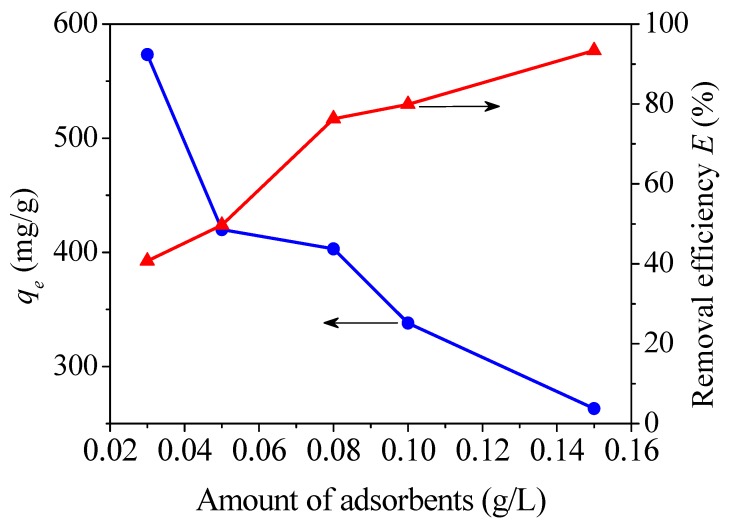
Effect of dosage with CoFe_2_O_4_@SiO_2_-Ppy as adsorbent.

The capacity and efficiency (*E*) were investigated by changing the dosage of CoFe_2_O_4_@SiO_2_-Ppy of 3, 5, 8, 10, and 15 mg with 100 mL 40 mg/L Hg^2+^ solution. As illustrated in Figure 11, as the dosage increases, the adsorption capacity shows a downward trend, but the removal efficiency increases. The dosage of 0.05 g/L is selected for subsequent test conditions.

#### 3.2.3. Influence of Adsorption Time

**Figure 12:**
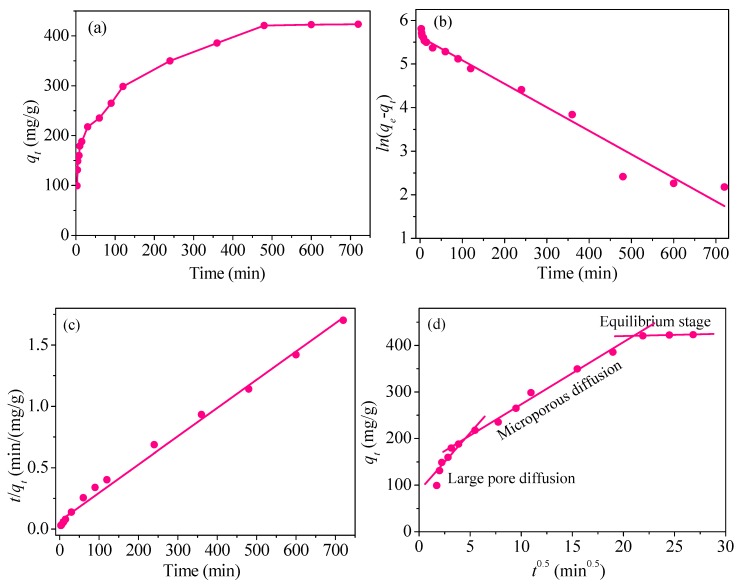
(**a**) Effect of contact time; results of (**b**) pseudo-first-order, (**c**) pseudo-second-order and (**d**) intraparticle diffusion.

As shown in Figure 12a, adsorption capacity increases over time, but the growth rate is different in different periods. In the first hour, the growth of adsorption capacity is significantly fast and reaches the half of the adsorption equilibrium. The reason is that the adsorbent is in the form of powder with the particle diameter of 90–140 nm, so the distance from mercury ions to the surface active site of the adsorbent becomes shorter. In addition, the large BET value of CoFe_2_O_4_@SiO_2_-Ppy provides lots of active sites for Hg^2+^. Simultaneously, the concentration gradient of Hg^2+^ between the solution and on the surface of CoFe_2_O_4_@SiO_2_-Ppy is enough large, resulting in a quick gathering of Hg^2+^ onto CoFe_2_O_4_@SiO_2_-Ppy.

Subsequently, with the reducing amount of available active sites and the concentration gradient, the adsorption rate slows. The adsorption equilibrium is reached after 8 h.

### 3.3. Adsorption Kinetics

To explore the possible reaction mechanism of CoFe_2_O_4_@SiO_2_-Ppy, the pseudo-first-order, pseudo-second-order, and intraparticle diffusion kinetics models were employed to fit the test results. The pseudo-first-order:

(1) $$\ln\left(q_{e}-q_{t}\right)=\ln q_{e}-k_{1}t$$

The pseudo-second-order:

(2) $$\frac{t}{q_{t}}=\frac{1}{q_{e}^{2}k_{2}}+\frac{t}{q_{e}}$$

The intraparticle diffusion:

(3) $$q_{t}=k_{di}t^{0.5}+C_{i}$$

here, *k*_1_ (min^−1^) and *k*_2_ (g/(mg·min)) are rate coefficient; *k_di_* (mg/(g·min^0.5^)) represents diffusion rate coefficient. *C_i_* (mg/g) is the thickness of the boundary layer. The test results were linearly fitted using the above three kinetic models and were shown in Figure 12b–d, and all the relevant results are listed in Table 2.

Regression coefficient (*R*^2^) in Figure 12b,c shows that pseudo-second-order fitting has a higher *R*^2^ compared with pseudo-first-order fitting with *R*^2^. Moreover, according to the calculated adsorption capacity (*q*_e,cal_) in Table 2, the *q*_e,cal_ in the pseudo-first-order fitting and pseudo-second-order fitting are 277.2 mg/g and 434.8 mg/g, respectively. The latter value is closer to the value *q*_e,exp_ of 420.8 mg/g, indicating that pseudo-second-order fitting is more consistent with the adsorption process.

The adsorption process was fitted by the intraparticle diffusion model and plotted in Figure 12d. From Figure 12d, the adsorption process consists of three different adsorption phases: large pore diffusion phase, microporous diffusion phase, and equilibrium adsorption phase. At the first phase, the adsorption rate is the fastest; at the second phase, the rate becomes relatively slow, and tends to be gentle at the final phase of adsorption.

For further comparison of each linear fitted stage, the values of *k_di_* and regression coefficients *R*^2^ in each stage were calculated separately and listed in Table 2. Obviously, the coefficients *k_di_* are in the order of *k_d_*_1_ > *k_d_*_2_ >> *k_d_*_3_, so the overall adsorption process with CoFe_2_O_4_@SiO_2_-Ppy as the adsorbent is mainly controlled by the first and second stages.

In the first stage, the concentration of Hg^2+^ is high and Hg^2+^ can quickly come into contact with the adsorbents. Numbers of unoccupied active sites provide favourable conditions for rapid adsorption. At the second stage, after almost all of the external activity sites are occupied, residual Hg^2+^ ions enter into the pores of CoFe_2_O_4_@SiO_2_-Ppy and then adsorb onto the inner surface of the pores [40]. Moreover, the adsorption capacity reaches 420.8 mg/g and approaches the adsorption equilibrium at the second stage.

Finally, the *k*_*d*3_ value of 0.49 mg/(g·min^0.5^) represents a state of near-adsorption equilibrium. The *R*^2^ obtained by the intraparticle diffusion model are not high, and the fitting line deviates from the origin, showing there are many factors existing during the process of adsorption.

### 3.4. Adsorption Isotherms

For the aim of further investigating the adsorption capacity of CoFe_2_O_4_@SiO_2_-Ppy on Hg^2+^, the experimental data was treated by the Langmuir (Equation (4)) and Freundlich (Equation (5)) isotherms.

(4) $$\frac{C_{e}}{q_{e}}=\frac{C_{e}}{Q_{m}}+\frac{1}{Q_{m}K_{L}}$$

here, *Q*_m_ and *K*_L_ represent maximum capacity (mg/g) and constant, respectively. The separation constant *R_L_* can be used to represent that the type of isotherms [20].

(5) $$lnq_{e}=lnK_{F}+\frac{1}{n}lnC_{e}$$

The separation factor:

(6) $$R_{L}=\frac{1}{1+K_{L}C_{0}}$$

here, *K*_F_ and *n* represent the Freundlich constants. 1/*n* represents the uneven factor, commonly used to describe the deviation degree of the adsorption linearity.

**Figure 13:**
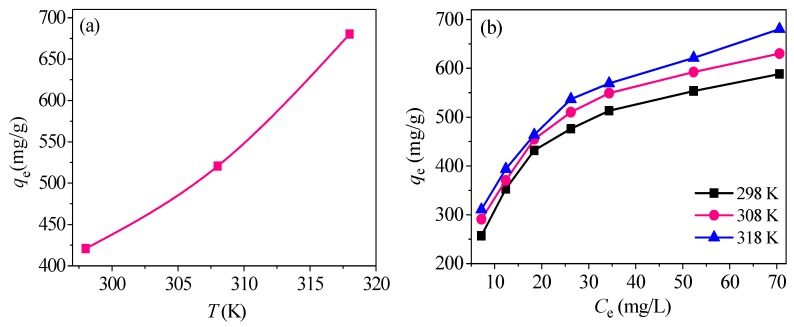

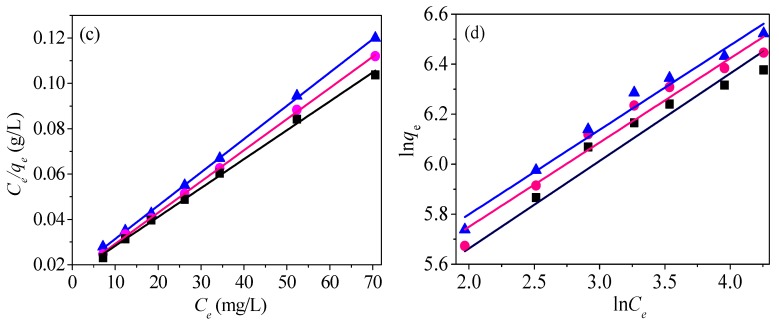
(**a**) Adsorption of Hg^2+^ under different temperatures; (**b**) adsorption isotherms; (**c**) Langmuir and (**d**) Freundlich isotherms of CoFe_2_O_4_@SiO_2_-Ppy.

The fitting results of two isotherm models are shown in Figure 13. The values of isothermal constant and *R*^2^ for Langmuir and Freundlich are listed in Table 3. *R*^2^ from Langmuir are over 0.99 and higher than Freundlich, indicating that the Langmuir fitting has good consistency with Hg^2+^ adsorption and the adsorption process is a molecule layer reaction. Moreover, chemical reaction may be the main effect factor [10]. *R_L_* from Langmuir is between 0 and 1, illustrating a favorable isotherm.

The calculated *Q*_m_ in the Langmuir model is 680.2 mg/g, much bigger than many other materials (Table 4). 1/n values from the Freundlich isotherm are all less than 0.35, indicating that relatively high adsorption intensity occurred [10].

By comparison, the Freundlich model has a poor fitting degree with *R*^2^ below 0.98. Therefore, the adsorption process of CoFe_2_O_4_@SiO_2_-Ppy for Hg^2+^ is more suitably depicted by the Langmuir model.

### 3.5. Adsorption Thermodynamics

**Figure 14:**
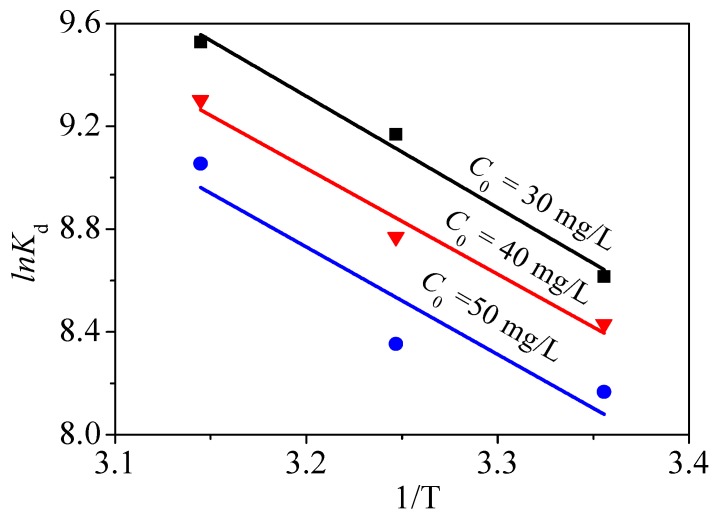
Linear fitting of thermodynamics.

Thermodynamic parameters of Gibbs free energy (Δ*G*^0^, kJ/mol), enthalpy (Δ*H*^0^, kJ/mol) and entropy (Δ*S*^0^, kJ/(mol·K)) can be used to analyze the thermodynamics based on the following equations:

(7) $$\Delta G^{0}=-RTlnK_{d}$$

(8) $$lnK_{d}=\frac{\Delta S^{0}}{R}-\frac{\Delta H^{0}}{RT}$$

here, R is 8.314 J/(mol·K). *K_d_* represents thermodynamic constant. Data obtained by *lnK_d_* versus 1/*T* is plotted and fitted to calculate Δ*H*^0^ and Δ*S*^0^ based on the slopes and intercepts of fitted plot. The results are exhibited in Figure 14 and Table 5.

The three positive Δ*H*^0^ values suggest that Hg^2+^ removal is endothermic. Negative Δ*G*^0^ values indicate a spontaneous adsorption and some chemical processes are involved [10], which is consistent with the analysis from adsorption isotherms. The positive Δ*S*^0^ values illustrate that a disorderly solid–solution interface and high temperature are favorable to the removal of Hg^2+^ by CoFe_2_O_4_@SiO_2_-Ppy [10,48].

### 3.6. Effect of Coexistence Ions

Natural water or industrial wastewater commonly contains various metal ions. These metal ions can affect on the adsorption of mercury through competing with Hg^2+^ for adsorption. Consequently, it is necessary to use CoFe_2_O_4_@SiO_2_-Ppy to survey the aggressive effect of ionic strength and coexisting ions on the ability of CoFe_2_O_4_@SiO_2_-Ppy.

**Figure 15:**
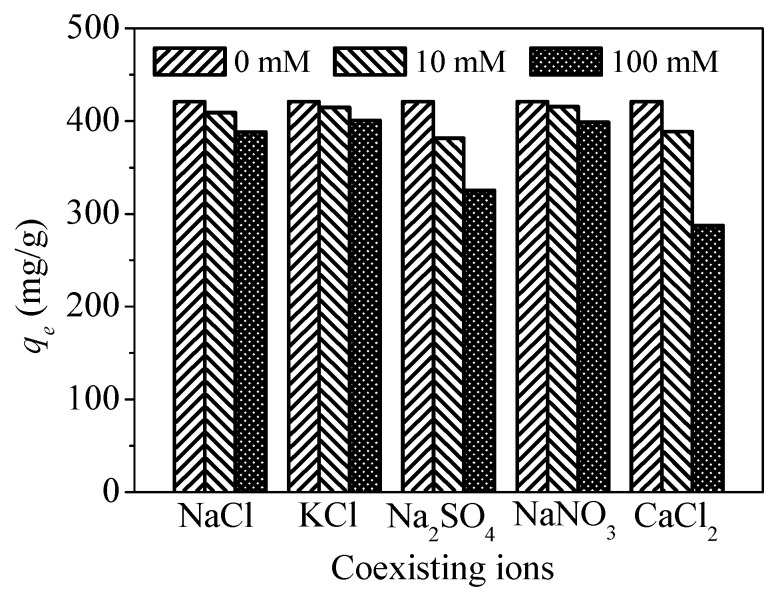
Effect of coexisting ions.

One hundred mL Hg^2+^ solution containing six common ions (Cl^−1^, NO_3_^−^, SO_4_^2−^, Na^+^, K^+^, and Ca^2+^) with different concentrations (0 mM, 10 mM, and 100 mM) was contacted with CoFe_2_O_4_@SiO_2_-Ppy (5 mg) at pH = 8 for 8 h. After the reaction, the residual content of Hg^2+^ was measured, and the corresponding data are shown in Figure 15.

As ionic concentration increases, the adsorption capacity of CoFe_2_O_4_@SiO_2_-Ppy for Hg^2+^ decreases. Among the three anions (Cl^−1^, NO_3_^−^, SO_4_^2−^), SO_4_^2−^ has a greater impact on the removal of Hg^2+^, and the removal efficiencies decrease by 9.29% and 22.74% at the concentrations of 10 mM and 100 mM, respectively. It may be because the amino group has a higher affinity for SO_4_^2−^ than Cl^−1^ and NO_3_^−^ [22].

Among these cations (Na^+^, K^+^, Ca^2+^), Ca^2+^ generates a large influence on the adsorption, and the capacity for Hg^2+^ removal is reduced by 13.12% and 31.73% at the concentrations of 10 mM and 100 mM, respectively. It may be because Ca^2+^ is a divalent cation and occupies two active adsorption sites [49].

### 3.7. Application Evaluation

In practical application, the adsorption and desorption performances are two key indices for judging an adsorbent. An ideal adsorbent should have a high absorbability. In addition, it is also important to have a good regeneration capacity, so that the material can be reused many times, thus greatly reducing the disposal cost.

**Figure 16:**
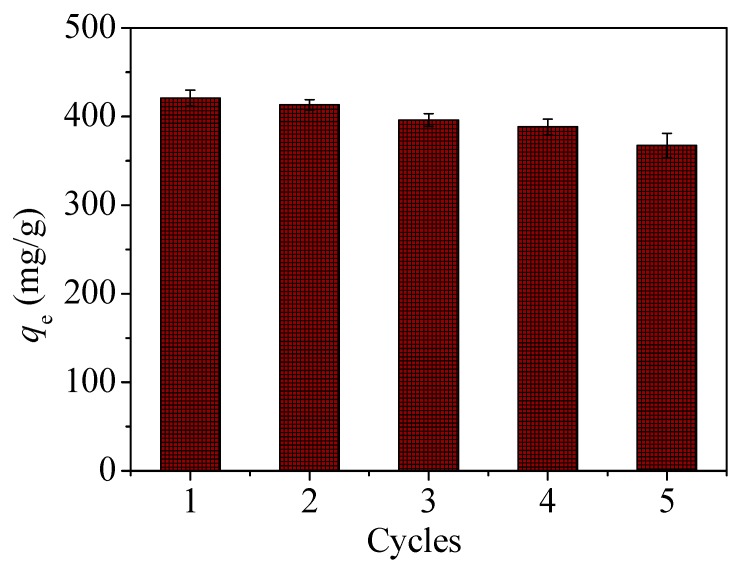
Adsorption and regeneration cycles of CoFe_2_O_4_@SiO_2_-Ppy.

It can be seen from the above experiments that changes in pH significantly affect the adsorption process of CoFe_2_O_4_@SiO_2_-Ppy for Hg^2+^. As pH increases, the adsorption capacity increases in the pH range 3–9. Thus, the desorption of CoFe_2_O_4_@SiO_2_-Ppy can be achieved by pickling with an acidic solution [48]. 0.005 g of CoFe_2_O_4_@SiO_2_-Ppy was first contacted with 100 mL Hg^2+^ solution (40 mg/L) for 8 h at 298 K. The resulting Hg^2+^-adsorbed CoFe_2_O_4_@SiO_2_-Ppy composite was filtered and then eluted with 100 mL 0.2 mol/L HCl solution. The whole process was repeated 5 times. The experimental results are exhibited in Figure 16.

After five cycles, the capacity of CoFe_2_O_4_@SiO_2_-Ppy for Hg^2+^ only decreased by 12.7% and still reached 367.3 mg/g. This shows that CoFe_2_O_4_@SiO_2_-Ppy is a promising heavy metal adsorption material.

To further assess the performance of CoFe_2_O_4_@SiO_2_-Ppy, electroplating wastewater was used as the target to be processed. In the test, the employed metal ions in the electroplating wastewater included Hg^2+^ (2.2 mg/L), Cr^3+^ (3.2 mg/L), Ni^2+^ (2.3 mg/L), Cu^2+^ (0.9 mg/L), and Cd^2+^ (2.5 mg/L). The Chemical Oxygen Demand was 76.6 mg/L. The used amount of adsorbent was 0.1 g/L.

The result shows that the efficiency *E* (%) achieved over 99.6% and the residual content of Hg^2+^ ions was below 0.05 mg/L, meeting the effluent standard of “Emission Standard of Pollutants for Electroplating” (GB 21900-2008). Based on the applied result, it has been demonstrated that CoFe_2_O_4_@SiO_2_-Ppy is a valuable and promising adsorbent.

### 3.8. Mechanism Speculation

It is well known that mercury has a variety of forms in aqueous solutions, including Hg^2+^, HgOH^+^, HgCl^+^, and Hg(OH)_2_ [48], etc. Under acidic conditions (pH < 3), mercury in solution is mainly in the forms of Hg^2+^, HgOH^+^, and Hg(OH)_2_ [48]. The formation of Hg^2+^ is the main morphology as the pH increases, and dissolved Hg(OH)_2_ gradually becomes the main morphology when pH is more than 6 [13].

Adsorption of Hg^2+^ with CoFe_2_O_4_@SiO_2_-Ppy is a process affected by pH. As the pH increases, the effect becomes greater. HgOH^+^, HgCl^+^, and Hg(OH)_2_ are abundant in the solution under alkaline conditions, and these ions are more easily adsorbed onto CoFe_2_O_4_@SiO_2_-Ppy due to their better size and higher mobility compared to Hg^2+^ [50].

The main adsorption site of Hg^2+^ is the N atom in the polypyrrole chain. Heavy metal ions can share solitary electrons with the N atom in the -N=C- group [51], as the N atom has a pair of electrons, which can form a complex with Hg^2+^ ions.

When pH < 5, the pair of electrons on the nitrogen is slightly protonated, hindering the formation of complexes. When the pH is at the range of 5–10, the main form of mercury is dissolved Hg(OH)_2_, able to form a stable structure of the complex with the pair of electrons on the nitrogen, causing a high removal of Hg^2+^ by CoFe_2_O_4_@SiO_2_-Ppy.

**Figure 17:**
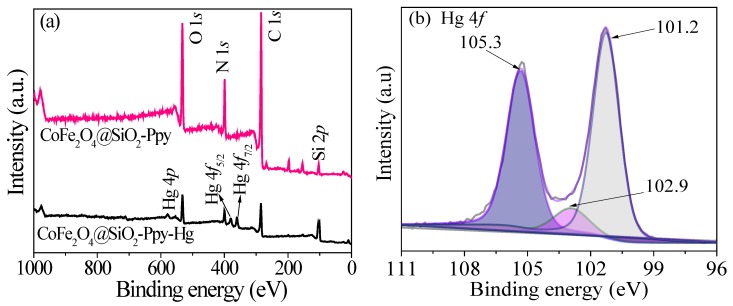

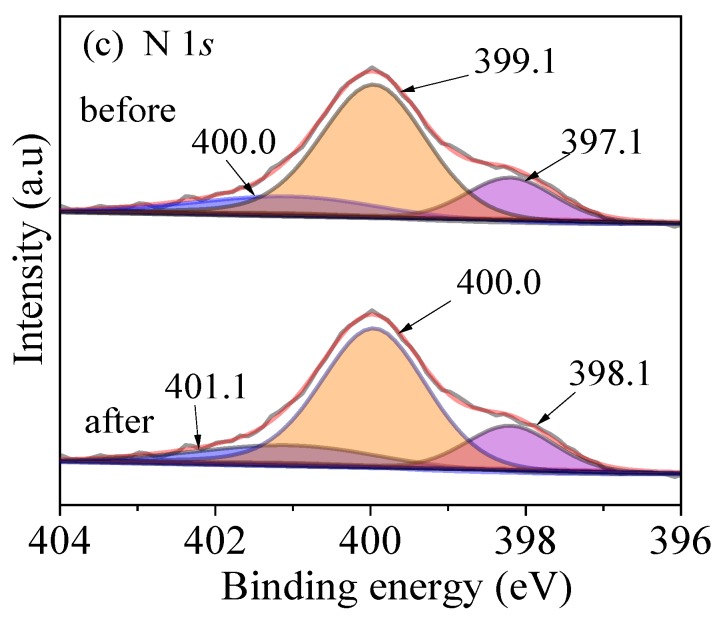
XPS patterns of (**a**) survey scan, (**b**) Hg 4*f* and (**c**) N 1*s*.

Figure 17a shows the XPS patterns of CoFe_2_O_4_@SiO_2_-Ppy. After adsorption, the intensities of C 1*s*, N 1*s*, O 1*s*, and Si 2*p* in CoFe_2_O_4_@SiO_2_-Ppy-Hg are reduced and new Hg 4*f* and Hg 4*p* appear. There are two peaks at 101.2 and 105.3 eV in Figure 17b, attributed to Hg 4*f*_5/2_ and Hg 4*f*_7/2_, respectively, and another peak at 102.9 eV is Si 2*p* [13]. In Figure 17c, due to the adsorption of Hg^2+^, the whole of the N peak is shifted and the shifted value is approximately at 1 eV [52].

The resluts indicates a chmical reaction is involved in the adsorption of Hg^2+^ onto CoFe_2_O_4_@SiO_2_-Ppy, which is consistent with the isotherm and thermodynamic analyses. XPS analysis directly proves that Hg^2+^ ions have been successfully attached to the surface of CoFe_2_O_4_@SiO_2_-Ppy.

## 4. Conclusions

A new polypyrrole-grafted magnetic compound, CoFe_2_O_4_@SiO_2_-Ppy, was successfully synthesized with a facile hydrothermal method under relatively safe conditions. CoFe_2_O_4_@SiO_2_-Ppy can effectively adsorb Hg^2+^ ions from water. The fittings of kinetics, isotherms, and thermodynamics showed the adsorption of Hg^2+^ was endothermic and spontaneous, and involved some chemical reactions. The value of *Q*_m_ from the Langmuir model reached 680.2 mg/g, exceeding that of many adsorbents. In addition, CoFe_2_O_4_@SiO_2_-Ppy had excellent regeneration ability, dispersibility, and stability. The application results show that CoFe_2_O_4_@SiO_2_-Ppy can be an excellent adsorbent for removing heavy metal ions from aqueous solutions.

## Figures and Tables

**Table 1:** Structure of three adsorbents.

Samples	BET (m^2^/g)	Total Pore Volume (cm^3^/g)	Pore Diameter (nm)
CoFe_2_O_4_	48.49	0.424	3.413
CoFe_2_O_4_@SiO_2_	225.36	0.552	3.062
CoFe_2_O_4_@SiO_2_-Ppy	218.56	0.888	3.106

**Table 2:** Kinetic fitting results of Hg^2+^ onto CoFe_2_O_4_@SiO_2_-Ppy.

**Pseudo-First-Order**								**Pseudo-Second-Order**						
*q*_e,exp_ (mg/g)	*q*_e,cal_ (mg/g)			*k*_1_ (1/min)		*R* ^2^		*q*_e,cal_ (mg/g)			*k*_2_ (g/(mg·min))		*R* ^2^	
420.8	277.2			0.0054		0.970		434.8			0.00008		0.993	
**Intraparticle Diffusion**														
*k*_d1_ (mg/(g·min^0.5^))		*C*_1_ (mg/g)	*R*_1_^2^		*k*_d2_ (mg/(g·min^0.5^))		*C*_2_ (mg/g)		*R*_2_^2^	*k*_d3_ (mg/(g·min^0.5^))		*C*_3_ (mg/g)		*R*_3_^2^
23.46		94.26	0.933		13.17		141.74		0.975	0.49		410.18		0.949

**Table 3:** Isotherm data of CoFe_2_O_4_@SiO_2_-Ppy.

T (K)	Langmuir Isotherm				Freundlich Isotherm		
	*Q*_m_ (mg/g)	*K*_L_ (L/mg)	*R* ^2^	*R*_L_	1/n	*K*_F_ (mg^1−n^ L^n^/g)	*R* ^2^
298	680.2	0.088	0.999	0.102	0.349	143.0	0.927
308	769.2	0.084	0.999	0.106	0.338	167.9	0.973
318	833.3	0.077	0.997	0.114	0.336	159.9	0.952

**Table 4:** Comparison of Hg^2+^ removal capability.

Adsorbent	pH	Fitting Models	*Q*_m_ (mg/g)	Ref.
Titanate nanotube adsorbents	10	Sips	140	[41]
Lignocellulosic	5	Langmuir	28	[42]
Modified magnetic chitosan	5	Langmuir	96	[43]
NH_2_-CoFe_2_O_4_-chitosan-graphene	7	Langmuir	361	[44]
functionalized Carbon nanotubes	5.5	Freundlich	186.97	[45]
Polypyrrole multilayer cellulose	6	Langmuir	31.68	[46]
Poly (2-aminothiazole)	6.5	Langmuir	325.7	[47]
CoFe_2_O_4_@SiO_2_-NH_2_	7	Langmuir	149.3	[10]
Short channel SBA-15-SH	8	Freundlich	195.6	[48]
CoFe_2_O_4_@SiO_2_-Ppy	8	Langmuir	680.2	This work

**Table 5:** Thermodynamic parameters.

*C*_0_	Δ*H*^0^	Δ*S*^0^	Δ*G*^0^		
			298 K	308 K	318 K
30	0.036	192.763	−21.346	−23.480	−25.422
40	0.035	184.635	−20.890	−22.457	−24.594
50	0.034	183.725	−20.235	−21.392	−23.941
